# Monitoring health diversity across the life span

**DOI:** 10.25646/9568

**Published:** 2022-03-30

**Authors:** Susanne Wurm

**Affiliations:** University Medicine Greifswald

Our health is not a static condition, but rather a dynamic equilibrium that people maintain and constantly re-establish in all phases of life, in constant interaction with their environment. This basic understanding of health has its roots in the founding preamble of the World Health Organization (WHO). This understanding of health has been known at least since the Ottawa Charter of the WHO (1986), but is not necessarily aware for many of us when ‘health’ is mentioned. We often continue to separate the two poles of health and illness. Chronic diseases and disabilities in particular are quickly associated with a static and thus permanent, unchangeable state.

However, research shows that chronic diseases can also be changed. Studies indicate, for example, that lifestyle changes can significantly lower high blood pressure and sustainably reduce type 2 diabetes [1, 2]; these are just two of many examples that are of great importance for our quality of life and life expectancy.

Our view of limitations and disabilities has also changed over the past decades. The international classification system ICIDH established by the WHO in 1980 described diseases, health impairments, disabilities and their social consequences (handicaps) in the form of a causal chain, as if this were an almost inevitable sequence. It was not until the turn of the millennium that the WHO initiated an important rethinking through the International Classification of Functioning, Disability and Health (ICF) by focusing more strongly on the dynamic equilibrium. The changed title of this classification already sends a signal, because disability and health are not mutually exclusive. Above all, the ICF makes it clear that health impairments do not necessarily limit activities and result in social impairments, but that this depends decisively on the interaction of environmental and personal factors. We can therefore do something – both as those affected by health impairments and in the role of the ‘environment’. The extent to which we support people in their need for independence, for example through private and professional assistance, through medical and technological progress, or through housing and urban development, is not only an important lever in preventing health impairments from becoming disabilities and social handicaps. It is also an expression of how humanly we shape our society.

The three Focus articles in the current issue of the Journal of Health Monitoring by Laura Krause, Franziska Prütz, Judith Fuchs and their co-authors raise awareness of health diversity across the life span by examining disabilities and health impairments in children and adolescents as well as in younger and older adults. All three contributions highlight the high need for prevention to avoid secondary diseases and problems. Regardless of their age, people with health challenges have a higher risk of developing further health problems, as exemplified by the findings on children’s oral health, depressive symptoms in adults, and lack of support for basic activities of daily living in old age. They all face particular risks because they have to draw on their psychological, social, financial or knowledge resources every day to a much greater extent than people without comparable health challenges. This puts them at greater risk of exhausting their resources. This is especially true for those who already have fewer resources.

In recent years, the studies of the RKI health monitoring and health reporting have become more diverse. Migration-sensitive methods of data collection have been established (Issue 3/2019 of the Journal of Health Monitoring, JoHM). The health of refugees (Issue 1/2021 of JoHM) and of lesbian, gay, bisexual and trans and intersex people was reported on (Special Issue S1/2020 of JoHM). The current issue provides an important complementary perspective on health diversity across the life span. Continuing these perspectives that have been started is a particular challenge. How can the most representative picture possible be obtained for those population groups that are difficult to recruit for studies? This is especially true for older people who need care and live in their own homes or in nursing homes. The COVID-19 pandemic has turned a magnifying glass on how difficult their living situation often is.

The WHO has declared the current decade as the Decade of Healthy Ageing, giving governments and societies four tasks: Older people must have access to good long-term care; older people should have access to all forms of health care, including prevention and health promotion; the physical and social environment, as well as the economic environment should become more age-friendly; and finally, negative age stereotypes, prejudices and discrimination against older people should be combated. These four goals require a special focus on the rapidly growing group of older people. If we replace ‘older’ with ‘all’ in all four goals, it becomes clear at the same time that achieving these goals will benefit diversity across the entire life span.

## References

[1] Roerecke M Kaczorowski J Tobe SW (2017) The effect of a reduction in alcohol consumption on blood pressure: a systematic review and meta-analysis. The Lancet Public Health 2(2):e108-e120 29253389 10.1016/S2468-2667(17)30003-8 PMC6118407

[2] Hallberg SJ Gershuni VM Hazbun TL (2019) Reversing Type 2 Diabetes: A Narrative Review of the Evidence. Nutrients 11(4) 10.3390/nu11040766 PMC6520897 30939855

